# A Geographically Widespread Outbreak Investigation and Development of a Rapid Screening Method Using Whole Genome Sequences of Enterohemorrhagic *Escherichia coli* O121

**DOI:** 10.3389/fmicb.2017.00701

**Published:** 2017-04-20

**Authors:** Ken-ichi Lee, Tomoko Morita-Ishihara, Sunao Iyoda, Yoshitoshi Ogura, Tetsuya Hayashi, Tsuyoshi Sekizuka, Makoto Kuroda, Makoto Ohnishi, EHEC Working Group in Japan, Hiroko Takenuma

**Affiliations:** ^1^Department of Bacteriology I, National Institute of Infectious DiseasesTokyo, Japan; ^2^Department of Bacteriology, Faculty of Medical Sciences, Kyushu UniversityFukuoka, Japan; ^3^Laboratory of Bacterial Genomics, Pathogen Genomics Center, National Institute of Infectious DiseasesTokyo, Japan; ^4^Local Public Health InstitutesJapan

**Keywords:** molecular typing, diarrhea, phylogeny enterohemorrhagic *Escherichia coli*, disease outbreak, single nucleotide polymorphism

## Abstract

From 2014 to 2015, we investigated a suspected nationwide outbreak of enterohemorrhagic *Escherichia coli* serogroup O121. However, similar pulsed field gel electrophoresis (PFGE) profiles and the lack of epidemiological links between the isolates made detection of the outbreak difficult. To elucidate a more precise genetic distance among the isolates, whole genome sequence (WGS) analyses were implemented in the investigation. The WGS-based single nucleotide polymorphism (SNP) analysis showed that 23 out of 44 isolates formed a distinct cluster (the number of intra-cluster SNPs was ≤8). Specific genomic regions in the clustered isolates were used to develop a specific PCR analysis. The PCR analysis detected all the clustered isolates and was suitable for rapid screening during the outbreak investigation. Our results showed that WGS analyses were useful for the detection of a geographically widespread outbreak, especially for isolates showing similar PFGE profiles and for the development of a rapid and cost-effective screening method.

## Introduction

Enterohemorrhagic *Escherichia coli* (EHEC) is a leading cause of foodborne illness worldwide that causes diarrhea, hemorrhagic colitis, and life-threatening hemolytic uremic syndrome (Pennington, 2010). More than 3,000 cases of infection are reported annually in Japan (Infectious Agents Surveillance Report, 2016), and the isolates are extensively monitored to detect nationwide outbreaks. All culture-positive cases of EHEC infection are reported to the National Institute of Infectious Diseases, Japan, irrespective of the serogroup. Currently, national surveillance of EHEC is performed using multilocus variable-number tandem-repeat analysis (MLVA) for serogroups O157, O26, and O111 (Izumiya et al., 2010) and pulsed field gel electrophoresis (PFGE) for the other serogroups.

Pulsed field gel electrophoresis can be used for the molecular typing of all EHEC serogroups and is available in many public health laboratories. Therefore, PFGE is regarded as the “gold standard” for the molecular typing of EHEC. However, PFGE has several drawbacks. The band recognition can be subjective, and a different threshold for a band position tolerance can generate different results. Additionally, fragments with the same size do not always have similar sequences and may not reflect the phylogeny (Noller et al., 2003; Li et al., 2009). Recently, molecular typing using whole genome sequence (WGS) analysis has become available. Phylogenetic analyses using single nucleotide polymorphisms (SNPs) or k-mer based methods from WGSs provide higher resolution for typing than the conventional typing methods. WGS typing has been widely used for various purposes, including EHEC outbreak surveillance (Grad et al., 2012), investigations of nosocomial infections of methicillin-resistant *Staphylococcus aureus* (Harris et al., 2013), and long-term tracing of *Clostridium difficile* in a community (Eyre et al., 2013). WGS typing is useful especially in outbreak investigations in which the isolates are highly clonal and difficult to distinguish using conventional methods (Salipante et al., 2015; Bekal et al., 2016). A recently developed network sharing WGS database has enabled more effective tracing of foodborne pathogens at the national and international levels, such as Genome Trakr Network (Allard et al., 2016) and EnteroBase^1^. Moreover, gene information specific to an outbreak strain can be extracted simultaneously (Grad et al., 2012; Sekizuka et al., 2015).

EHEC O121 is one of the most common non-O157 serogroup. In Japan, 12–96 isolates were reported annually during the past decade, which correspond to 0.6–4.4% of all EHEC cases^2^. We detected several EHEC O121 isolates showing high PFGE similarity in the national surveillance program from 2014 to 2015. Because most of the isolates did not have epidemiological links with other isolates and because PFGE did not have sufficient discriminatory power for the isolates, WGS typing was used to identify links between the isolates. Furthermore, we developed a rapid and cost-effective screening method for many samples to detect outbreak strains using WGS information.

## Materials and Methods

### EHEC O121 Isolates Used in This Study

From June 2014 to July 2015, EHEC O121 isolates that showed similar PFGE profiles were identified from the national EHEC surveillance. Personal information of the patients was completely anonymized and only the information of isolation date, isolation site and symptoms was used in this study. All but one isolate (141341, isolated from an asymptomatic carrier) were isolated from patients that showed abdominal pain, diarrhea or hemolytic uremic syndrome and their family. Five isolates (150337–150341) were obtained from the same family. However, there was no clear epidemiological link in the other isolates. Forty-one isolates (140961–151387) that formed a cluster with more than 85% PFGE similarity were used for the WGS analyses (**Table 1**). Additionally, three previously collected isolates (121512, 132137, and 140452) were used. The serial isolate numbers provided in **Table 1** were used throughout this study. The genes for Shiga toxin (*stx*) and their subtypes were determined by PCR (Scheutz et al., 2012).

**Table 1:** Strain information used in this study.

ID	Serial no. in this study	Isolation date	Prefecture	*stx* subtype	MLST
121512	1	2008-07-24	Okayama	2a	655
132137	2	2013-09-27	Shiga	2a	655
140452	3	2014-03-13	Niigata	2a	655
140961	4	2014-06-28	Gumma	2a	655
140990	5	2014-06-25	Ishikawa	2a	655
141004	6	2014-06-30	Chiba	2a	5536
141202	7	2014^a^	Shizuoka	2a	655
141247	8	2014-07-30	Osaka	2a	5536
141341	9	2014-07-09	Hiroshima	2a	655
141544	10	2014-07-24	Gumma	2a	655
142136	11	2014-08-29	Miyagi	1a,2a	655
142321	12	2014-08-27	Kagoshima	2a	655
142478	13	2014-10-09	Yamagata	2a	655
142676	14	2014-12-01	Osaka	2a	655
142774	15	2014-12-04	Fukuoka	2a	655
150151	16	2014-12-03	Ibaraki	2a	655
150152	17	2015-01-13	Ibaraki	2a	655
150174	18	2014-08-25	Fukuoka	2a	655
150213	19	2014-12-11	Shizuoka	2a	655
150238	20	2014-12-07	Miyagi	2a	655
150239	21	2014-12-17	Miyagi	2a	655
150240	22	2014-12-22	Miyagi	2a	655
150241	23	2014-12-19	Miyagi	2a	655
150242	24	2014-12-19	Osaka	2a	655
150243	25	2014-12-24	Osaka	2a	655
150281	26	2014-12-15	Nara	2a	655
150337	27	2015-01-28	Nagano	2a	655
150338	28	2015-01-28	Nagano	2a	655
150339	29	2015-01-31	Nagano	2a	655
150340	30	2015-01-31	Nagano	2a	655
150341	31	2015-02-01	Nagano	2a	655
150342	32	2014-06-30	Kanagawa	2a	655
150373	33	2014-12-19	Yamagata	2a	655
150375	34	2014^a^	Tokyo	2a	655
150376	35	2014^a^	Tokyo	2a	655
150387	36	2014-12-24	Aomori	2a	655
150393	37	2014^a^	Shizuoka	2a	655
150395	38	2015^a^	Hyogo	2a	655
150400	39	2014-12-10	Miyazaki	2a	655
150542	40	2014-12-26	Okayama	2a	655
150616	41	2015-03-31	Yamagata	2a	655
150977	42	2015-05-21	Kagawa	2a	655
151171	43	2015-06-17	Kanagawa	2a	655
151387	44	2015-07-21	Kanagawa	2a	655


*^a^Isolation date is not available.*

### PFGE and MLST Analyses

Pulsed field gel electrophoresis was performed as described elsewhere (Pei et al., 2008), with minor modifications. In brief, bacterial cells on an agar medium were suspended in 200 μl of distilled water, and the samples were mixed with an equal amount of 1% SeaKem Gold agarose (Lonza, Basel, Switzerland) to induce plug formation. After appropriate preparations for restriction endonuclease digestion, the DNA in each plug was digested with 30 U of *Xba*I (Roche Diagnostics, Basel, Switzerland) at 37°C for 2.5 h. The PFGE was performed using a CHEF DRIII system (Bio-Rad Laboratories, Hercules, CA, USA) with the following run parameters: a switch time of 2.2–54.2 and a run time of 21 h. *Salmonella enterica* serovar Braenderup H9812 was used as the size standard. Analysis in BioNumerics 6.6 (Applied Math, Kortrijk, Belgium) was performed with a 1% position tolerance. An unweighted pair group method with arithmetic mean (UPGMA) clustering algorithm was used to create a hierarchical dendrogram, and the genetic similarity between the isolates was calculated using the Dice coefficient.

### WGS Analyses for the Phylogenetic Tree and Pairwise SNP Distances

Whole genome sequences were obtained using MiSeq (Illumina, San Diego, CA, USA). The genomic DNA libraries were prepared using a Nextera XT DNA sample prep kit (Illumina). The pooled libraries were subjected to multiplexed paired-end sequencing (300 mer × 2). The sequence reads were assembled using the A5-miseq pipeline (Coil et al., 2015). The contig sequences were aligned with the contigs of isolate 1 using MUMmer version 3.2259 (Kurtz et al., 2004) to identify the conserved backbone (core genome) of these strains and the SNP sites. A 4,645,249 bp sequence was conserved in all the strains examined, with >99% sequence identity and a >2,000 bp alignment length. The recombinogenic regions were removed by RecHMM (Zhou et al., 2014). Phylogenetic relationships were determined by reconstructing a phylogenetic tree using the maximum likelihood method based on the Tamura-Nei model with 1,000 bootstraps (Tamura and Nei, 1993) using the MEGA 7 software (Kumar et al., 2016). For the clustered isolates, a median joining network tree was constructed using the PopART software ver. 1.7^3^. The phylogenetic tree was also visualized with a partial national map of Japan using GenGIS software version 2.5.1 (Parks et al., 2013). In the map, each isolate was plotted on the prefectural capital of the isolation site. To confirm that the clustered isolates were genetically distinct from the other isolates, a root-to-tip analysis was performed. The root-to-tip distance of the clustered isolates and the most closely related isolate 18 were calculated using the Path-O-Gen software (Rambaut et al., 2016).

### *In Silico* Analyses of the Draft Genomes

Three molecular typing schemes were applied to the contigs assembled as described above. Multilocus sequence typing (MLST) was performed according to the protocols available in the *E. coli* MLST database^4^ (Wirth et al., 2006). Inc type of the plasmids were investigated using PlasmidFinder 1.3^5^.

### Development of a Plausible Outbreak-specific Screening Method Using PCR

**FIGURE 1:**
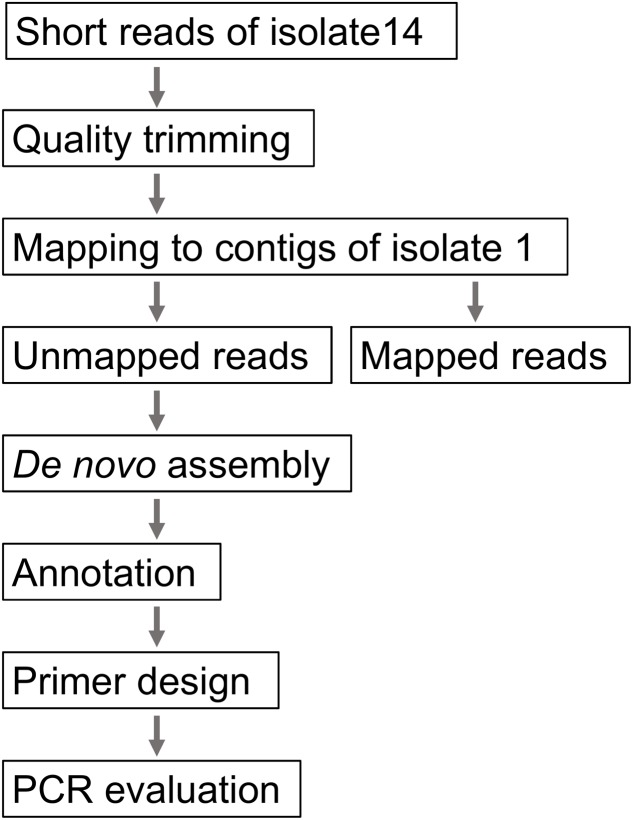
**Procedure for the development of a plausible outbreak-specific screening method using PCR**.

The WGS analyses identified a cluster consisting of 23 isolates. We regarded this cluster as a plausible outbreak cluster. A rapid screening method using a PCR assay was developed in preparation for future outbreaks by the isolate and to trace the contaminated food product (**Figure 1**). First, short reads of one outbreak-associated isolate (isolate 14) were mapped to the contigs of isolate 1 using the CLC genomics workbench (QIAGEN, Inc., Valencia, CA, USA). Second, the unmapped reads were subjected to *de novo* assembly, and the generated contigs were annotated using the Microbial Genome Annotation Pipeline (MiGAP^6^) annotation server. Third, two pairs of primers [HP-1 (5′-CGTTTGGCATACTGGGTTGC-3′) and HP-2 (5′-GTCTGACCAGAGCTCGCTTT-3′), which generate a 288 bp amplicon, and HP-5 (5′-TTTACATGGCGGGGAATCGT-3′) and HP-6 (5′-CCTGCACCCACCGTTCATAA-3′), which generate a 609 bp amplicon] were constructed from the specific regions. The specificity and sensitivity were evaluated using all the O121 isolates described above and 11 EHEC isolates belonging to other serogroups (O157, 1 isolate; O26, 10 isolates; O103, 4 isolates; O145, 4 isolates; and O165, 4 isolates).

### Accession Number

The FASTQ sequences and assembled contigs used in this study were deposited in the DNA Data Bank of Japan^7^ under accession number DRA005295.

## Results

### PFGE, MLST and Plasmid Replicon Typing

**FIGURE 2:**
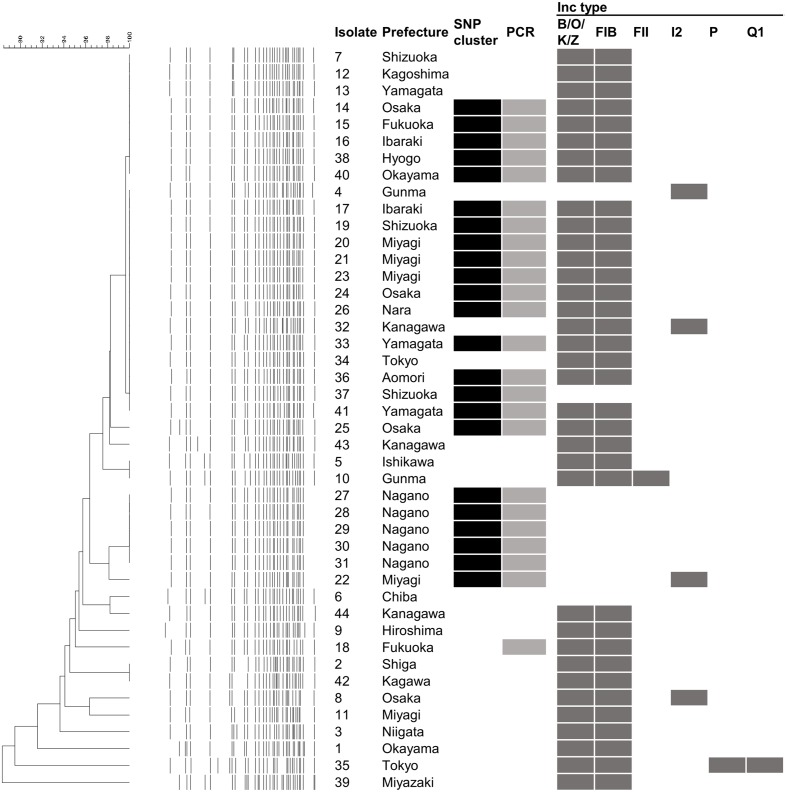
**Results of pulsed field gel electrophoresis (PFGE), whole genome sequence (WGS)-based phylogenetic analysis WGS-based PCR and *in silico* plasmid replicon typing.** The isolate denotes the serial number in **Table 1**. The dendrogram and electrophoretic profiles were obtained by PFGE of *Xba*I-digested DNA. The black and gray squares indicate clustered isolates in the WGS analysis and the detection of the specific amplicon by PCR, respectively. Dark gray squares indicate the presence of the corresponding Inc type plasmid.

According to PFGE results, two major clusters were generated at the 99% threshold of the Dice similarity coefficient (**Figure 2**). The first cluster consisted of 22 isolates. Although these isolates were isolated over a 1-year period, the isolation sites of some of the isolates were separated by a great distance (>1,000 km). The other major cluster consisted of five isolates that were isolated from a single family. However, 36 isolates, including the two clusters described above, formed one cluster at the 95% threshold. These isolates showing similar PFGE profiles were isolated over a 14-month period from a widespread area in Japan. Because no common epidemiologic factors linked these isolates, whether these isolates were derived from the same source was unclear. MLST did not provide sufficient resolution. All but two isolates belonged to ST 655. The other two isolates belonged to ST 5536, which had a one locus difference from ST 655 (**Table 1**). Plasmid replicon typing also did not have sufficient discriminatory power (**Figure 1**).

### Phylogenetic Analysis Using Whole Genome Sequences

**FIGURE 3:**
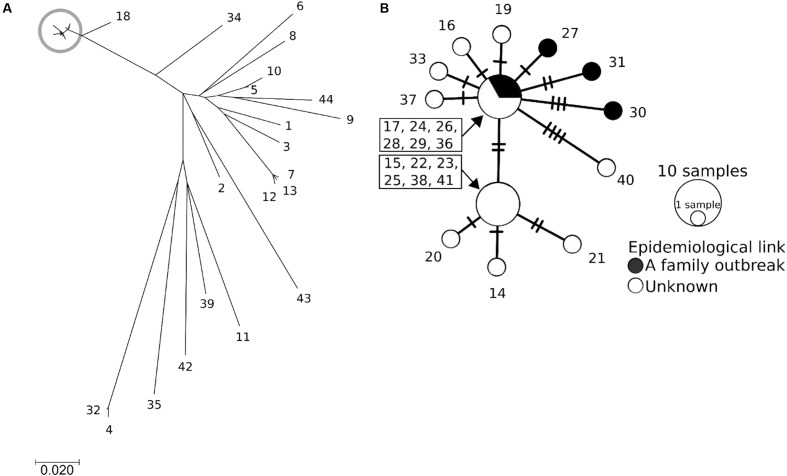
**The phylogenetic analysis results obtained using EHEC O121 whole genome sequences.** The isolate number denotes the serial number in **Table 1**. **(A)** Maximum likelihood tree. The scale bar shows the number of substitutions per site. The gray circle indicates the clustered isolates. **(B)** Median joining network generated from SNPs of the clustered isolates, which are shown in the gray circle in **(A)**. The sizes and colors of the circles represent the number of isolates and the epidemiological link between the isolates, respectively. A crossing bar on a connecting line shows one SNP locus.

**FIGURE 4:**
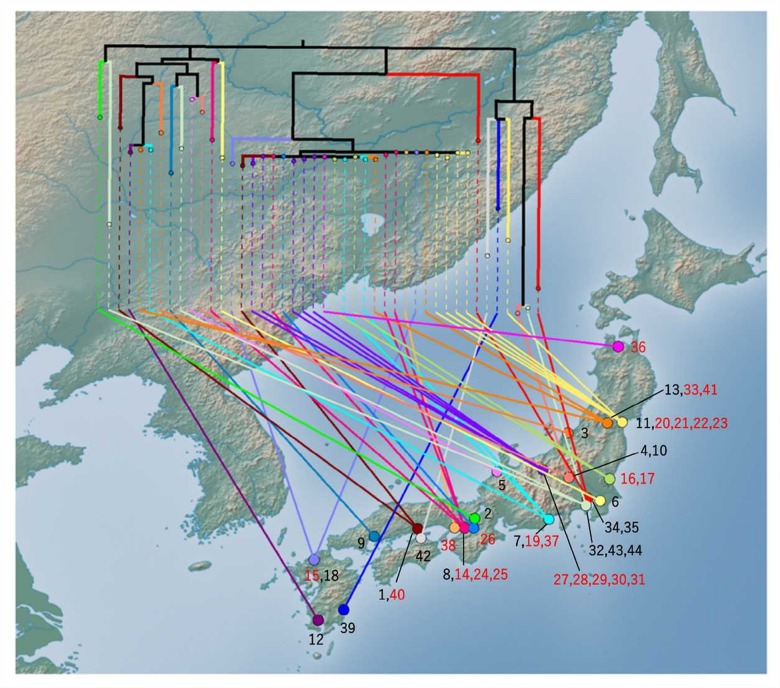
**Phylogenetic tree mapped onto the national map of Japan using GenGIS software.** The maximum likelihood tree was linked to the isolation site. Black branches in the tree indicate internal edges, which cover multiple prefectures. The isolation sites were mapped to the capital of each prefecture. The number represents the serial number of the isolate. The number of clustered isolates was indicated as red character.

**FIGURE 5:**
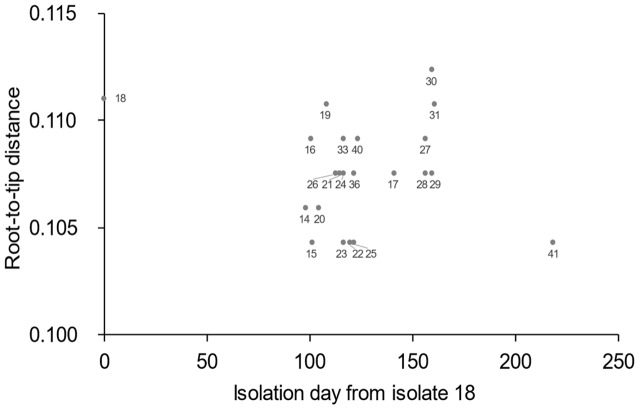
**Root-to-tip analysis results.** Values on the x-axis represent the isolation day with isolate 18 regarded as zero. The value on the y-axis represents the distance of each isolate from the root of the phylogenetic tree geneated using Path-O-Gen software.

To gain a more precise insight into a set of isolates with similar PFGE profiles, WGSs were obtained and SNPs were extracted from the core genome of the 44 isolates used in this study. The concatenated alignment of 713 SNP sites located in the conserved backbone was used for further analyses. The maximum likelihood tree indicated that 23 isolates formed a cluster (**Figure 3A**). In the cluster, the pairwise SNP distances ranged from zero to eight, with mean and median values of 2.6 and 2.0, respectively (**Figure 3B**). The cluster could be subdivided into two groups consisting of 14 (upper part of **Figure 3B**) and 9 (lower part of **Figure 3B**) isolates. The geographical location and date information for the isolates did not distinguish these two groups (**Figure 4**). One of the groups included isolates from the same family members, in which the maximum pairwise SNP distance was five. The most closely related isolate to the cluster was isolate 18 (the mean pairwise SNP was 17). The other isolates had 55 or more pairwise SNPs from the cluster. The root-to-tip analysis reinforced that the clustered isolates were genetically distinct from the other isolates (**Figure 5**). This analysis shows a proportionate relationship between isolation interval (x-axis) and distance from the root of the phylogenetic tree (y-axis) if the isolates were originated from the same ancestor, assuming a uniform mutation rate. Therefore, the isolates from the same ancestor will be plotted along a line. Isolates 18 and 41 were the outliers from the clustered isolates. The phylogenetic analysis results suggested that the clustered isolates and isolate 18 had different ancestors. However, isolate 41 was included in the cluster by the phylogenetic analysis, which suggested that bacterial multiplication might be inhibited in certain environments, such as food stored in low temperature.

### Development of a Plausible Outbreak-specific Screening Method Using PCR

To perform rapid and more precise screening for the clustered isolates, a WGS-based PCR method was developed. When short reads of isolate 14 were mapped to the contigs of isolate 1, 1.05% of the short reads (22,962/2,177,097 reads; average length = 216.0 bp) were not mapped. After *de novo* assembly of the unmapped reads and subsequent annotation, 42 contigs (>300 bp) were generated. Most of the contigs were derived from prophages or transposon sequences. Excluding these mobile genetic elements, we designed primer pairs to detect two coding sequence (CDS) regions that were specific to isolate 14. Both CDSs encoded hypothetical proteins that were highly unique to the clustered isolates. Two pairs of primers successfully generated a band of the expected size in the clustered isolates (**Figure 2**). In addition to these isolates, specific amplification was detected in isolate 18, which was closely related to the clustered isolates. No specific amplification was detected in the other O121 isolates or the isolates of the other serogroups.

## Discussion

Our study showed that molecular typing using WGS was useful for the outbreak investigation of EHEC isolates with high PFGE similarity. Regarding EHEC, O121 isolates often show higher PFGE similarity than other serogroups. Using national surveillance data of PFGE analyses over the last 5 years, O121 has shown the highest mean pairwise similarity in the following major O serogroups: O121, 90.3%; O157, 84.5%; O26, 86.6%; O111, 89.2%; O103, 76.8%; and O145, 81.9% (data not shown). This high PFGE similarity prompted us to use WGS data to subtype the isolates. Using WGS-based SNPs, we found a plausible epidemiological link between epidemiologically unlinked isolates. In this study, although further tracing of the causative agents or the source could not be conducted, the nationwide spread of highly clonal isolates (**Figure 4**) suggested that a widely distributed food could be contaminated with EHEC O121. The mean pairwise SNP distance among the isolates in the cluster (2.6) was smaller than the maximum SNP distance in a family outbreak (5) (**Figures 2**, **3B**). This finding also indicates that the isolates in the cluster are genetically indistinguishable. Previous studies of WGS typing from an outbreak investigation showed that EHEC isolates with a known epidemiological link had a distance of less than eight SNPs (Underwood et al., 2013; Joensen et al., 2014; Holmes et al., 2015). The pairwise SNP distances among the cluster in this study ranged from zero to eight and were concordant with previous studies, which suggested a plausible nationwide outbreak. However, interpretation of the pairwise SNP distances requires careful attention. The SNP distance cannot be simply compared because sequencing technology and analytical procedures can vary between studies. Additionally, the core genome size is affected by the sample size and the genetic heterogeneity of the samples. Therefore, the SNP distance should be interpreted with epidemiological information to detect the disease outbreak.

Although the method demonstrated high resolution, WGS-typing is in the trial implementation phase in many public health laboratories. To date, major implementation of WGS-typing is a complement to conventional methods. When the results obtained through WGS typing and conventional methods are compared, the differences in methodology should be considered. In our results, isolates with no SNPs showed several PFGE patterns. A difference in the PFGE pattern in the same SNP cluster can be explained by an insertion, deletion, and inversion (Iguchi et al., 2006), amplification of a genomic island (Lee et al., 2015), and the gain and loss of mobile genetic elements, such as plasmids, prophages and transposons. Indeed, *in silico* plasmid replicon typing showed that variation in the same cluster. Inc B/O/K/Z and FIB plasmids, which are carried by most of the clustered isolates, were not detected from the isolates 22, 27–31, and 37 (**Figure 1**). These differences in plasmids could affect the result of PFGE. Other mobile genetic elements and a large-scale change in genomic structure could not be detected from our short-read data. Long read data may further improve the discriminatory power.

In addition to high resolution phylogenetic analyses, WGSs have a wide range of applications, including extracting isolate-specific regions and *in silico* typing for serotypes, virulence factors and antimicrobial resistance genes (Joensen et al., 2014). In our study, we used WGSs to develop a simple PCR-based detection method for the clustered isolates. Our strategy using the unmapped reads (**Figure 1**) successfully detected the clustered isolates. Combining WGS-based phylogenetic analyses with an outbreak-specific detection method will be a promising strategy for outbreak investigations, especially outbreaks that are widespread or continue over a prolonged period. This rapid and cost-effective detection method is useful for screening many clinical or food samples to find the link to etiological agents.

## Conclusion

The WGS-based phylogenetic analysis revealed the clonal EHEC O121 isolates that could not be identified by conventional PFGE. The results suggest the occurrence of a nationwide outbreak by the clonal isolates. We also developed a WGS-based outbreak-specific detection method. With our strategy, once an outbreak cluster is identified, screening can be conducted by cost-effective PCR. This strategy can be applied to investigations of outbreaks caused by various pathogens.

## Author Contributions

KL, TM-I, SI, and MO performed the experiments and wrote the paper. KL, YO, TH, TS, and MK analyzed the data. EHEC Working Group collected the isolates.

## Conflict of Interest Statement

The authors declare that the research was conducted in the absence of any commercial or financial relationships that could be construed as a potential conflict of interest. The reviewer AMG and handling Editor declared their shared affiliation, and the handling Editor states that the process nevertheless met the standards of a fair and objective review.

## Footnotes

1 http://enterobase.warwick.ac.uk/

2 http://www.nih.go.jp/niid/en/iasr-vol37-e/865-iasr/6488-435te.html

3 http://popart.otago.ac.nz

4 http://mlst.warwick.ac.uk/mlst/dbs/Ecoli

5 https://cge.cbs.dtu.dk/services/PlasmidFinder/

6 http://www.migap.org/index.php/en

7 http://www.ddbj.nig.ac.jp
